# Predictive significance of Charcot‐Leyden crystal structures for nasal polyp recurrence

**DOI:** 10.1002/clt2.12212

**Published:** 2022-11-22

**Authors:** Wenyi Chen, Yurong Bai, Weifeng Kong, Xin Luo, Yinhui Zeng, Jingyuan Chen, Xinyue Wang, Qingwu Wu, Shuvam Chaudhuri, Jianning Chen, Qintai Yang, Yana Zhang

**Affiliations:** ^1^ Department of Otolaryngology‐Head and Neck Surgery The Third Affiliated Hospital of Sun Yat‐Sen University Guangzhou China; ^2^ Department of Otolaryngology‐Head and Neck Surgery Guangzhou Women and Children's Medical Center Guangzhou China; ^3^ Department of Pathology Northwestern University Feinberg School of Medicine Chicago Illinois USA; ^4^ Department of Pathology The Third Affiliated Hospital of Sun Yat‐Sen University Guangzhou China; ^5^ Department of Allergy The Third Affiliated Hospital of Sun Yat‐Sen University Guangzhou China

## Abstract

**Background:**

Charcot‐Leyden crystals (CLCs) are recognized to be classic hallmarks of eosinophilic inflammation. Both protein and mRNA levels of CLC in nasal secretions and nasal brushing samples have been associated with nasal polyp recurrence. However, whether the crystalline CLC structures in nasal tissue could serve as an effective biomarker to predict polyp recurrence remains unclear.

**Methods:**

A total of 110 patients with chronic rhinosinusitis with nasal polyps (CRSwNP) completing the postoperative follow‐up over a period of 24 months were recruited. Hematoxylin and eosin staining was employed for CLCs identification. The predictive factors for polyp recurrence were determined by binary logistic regression analysis.

**Results:**

Thirty three (30.00%) patients developed recurrence during a 24‐month postoperative follow‐up, in which 84.85% (28/33) patients had crystalline CLC structures. Logistic regression analysis showed that crystalline CLC structure in nasal tissues is predictive of polyp recurrence. Youden index demonstrated crystalline CLC structure higher than 1 per high power field can predict postoperative polyp recurrence with 84.80% sensitivity and 98.70% specificity.

**Conclusions:**

The crystalline CLC structures in nasal tissues may serve as an easy‐counting and promising biomarker to predict CRSwNP recurrence.

To the editor,

Chronic rhinosinusitis with nasal polyps (CRSwNP) is a heterogeneous and challenging inflammatory airway disease involving non‐eosinophilic and eosinophilic endotypes. In fact, 98.5% eosinophilic CRSwNP (Eos CRSwNP) patients were recurrent after undergone endoscopic sinus surgery (ESS).^1^ Therefore, the identification of predictors of recurrence for Eos CRSwNP is crucial for better disease control. The local tissue eosinophilia has been proven to be the most important predictor.^2^ However, there are no standard methods for evaluating tissue eosinophilia due to uneven distribution and diversity in geographic conditions.^2^, ^3^ Thus, there are limitations to the predict recurrence of CRSwNP based only on tissue eosinophilia.

Charcot‐Leyden crystals (CLCs), composed by Galectin‐10 (Gal‐10), were first proposed in the late 1800s by Charcot and Leyden. The typical CLCs are structures identified as needle‐shaped bipyramidal Gal‐10 crystals detected by morphological methods. Gal‐10 protein have three distinct forms including crystalline CLC/Gal‐10 crystal structures (CLCs), extracellular vesicles, and extracellular soluble Gal‐10.^4^ Recent studies have indicated that CLC mRNA and protein levels in nasal brushing/secretions/tissues detected by ELISA and PCR were used as predictive indicators for recurrent CRSwNP and glucocorticoid sensitivity.^5^, ^6^, ^7^ Since only crystalline CLCs, but not soluble CLC/Gal‐10 protein, drive type 2 immunity, allergy and neutrophilic inflammation,^8^, ^9^ the predictive value of functional CLCs for nasal polyp (NP) relapse should be evaluated. However, little is known regarding whether crystalline CLC structures can be utilized to predict NP recurrence.

This was a retrospective study using data collected from patients with bilateral NPs who had undergone ESS at The Third Affiliated Hospital of Sun Yat‐sen University from January 2016 to December 2016. The patients treated with oral/nasal glucocorticoids, immunomodulatory agents, or antibiotics with 1 month before enrollment were exclude, and patients with fungal sinusitis, cystic fibrosis, allergic fungal rhinosinusitis, or primary ciliary dyskinesia were excluded. Eosinophilic CRSwNP was defined when eosinophil percentage >10% of the total infiltrating cells as previously described.^10^ The current study, through the post‐operative follow‐up of CRSwNP patients for 24 months, detected presence of CLCs in NPs and hypothesized that crystalline CLC structures could predict the recurrence of NPs after ESS.

Overall, 33 (30.00%) out of 110 patients were identified by polyp recurrence, whereas 77 (70.00%) patients did not demonstrate any features of recurrence. The demographic and clinical data of the subjects are shown in Table 1. We found that patients from the recurrent group had increased comorbid rate of asthma (*p* = 0.015) compared with recurrent‐free group. In addition, the percentage of male gender is 84.85% in the recurrent group, which is much higher than that in recurrent‐free group (57.14%) (*p* = 0.005). Moreover, CLCs count (*p* < 0.001) and eosinophils count (*p* < 0.001) in nasal tissues and both percentage (*p* = 0.001) and count number (*p* < 0.001) of eosinophils in peripheral blood (PB) were significantly evaluated in recurrent group compared with recurrent‐free group.

**TABLE 1:** Demographic and clinical characteristics of recruited patients

Variables	Recurrence (*n* = 33)	Non‐recurrence (*n* = 77)	*p* value
Age (years)	39.00 [32.00.48.50]	44.00 [32.00.51.50]	0.495
Gender, male (%)	28 (84.85%)	44 (57.14%)	0.005
Allergic rhinitis (%)	4 (12.12%)	14 (18.18%)	0.431
Asthma (%)	9 (27.27%)	6 (7.79%)	0.015
N‐ERD (%)	0 (%)	0 (%)	‐
Atopy (%)	19 (57.58%)	33 (42.86%)	0.157
Smoking history (years)	5 (15.15%)	13 (16.88%)	0.822
VAS scores			
Nasal obstruction	6.00 [4.25.8.00]	5.50 [3.00.9.00]	0.929
Rhinorrhea	5.00 [2.25.7.75]	3.00 [1.00.5.25]	0.121
Facial pain/headache	0.00 [0.00.1.75]	0.00 [0.00.1.00]	0.739
Olfactory disorder	6.50 [4.25.9.75]	4.00 [1.75.9.00]	0.140
Eos (#) in NPs	93.20 [58.00, 198.50]	4.80 [3.00.9.80]	<0.001
CLCs (#) in NPs	13.00 [2.00.23.50]	0.00 [0.00.0.00]	<0.001
Eos (%) in PB	6.00 [3.25.8.55]	2.50 [0.90.5.05]	0.001
Eos (#) in PB	0.42 [0.23.0.59]	0.18 [0.07.0.30]	<0.001
Neu (%) in PB	54.00 [46.95.61.15]	54.90 [48.10.62.35]	0.708
Neu (#) in PB	3.80 [2.98.4.80]	3.78 [3.16.4.82]	0.858
CLCs presence			
CLCs + CRSwNP	28 (84.85%)	1 (1.30%)	<0.001
CLCs − CRSwNP	5 (15.15%)	76 (98.70%)	
Eos CRSwNP grouping			
Eos CRSwNP	29 (87.88%)	16 (20.78%)	<0.001
Non‐Eos CRSwNP	4 (12.12%)	61 (79.22%)	

Abbreviations: #, count number; CLCs, Charcot‐Leyden crystals; Eos CRSwNP, eosinophilic chronic rhinosinusitis with nasal polyps; Eos, eosinophil; N‐ERD, nonsteroidal anti‐inflammatory drugs‐exacerbated respiratory disease; Neu, neutrophil; non‐Eos CRSwNPs, non‐eosinophilic chronic rhinosinusitis with nasal polyps; NPs, nasal polyps; PB, peripheral blood; VAS, Visual Analogue Scale.

To evaluate the presence of CLCs in NP biopsies, H&E staining was performed. The bipyramidal crystalline CLC structures were present in 29 out of 110 (26.36%) patients with CRSwNP, and present in 64.44% patients with Eos CRSwNP (Figure 1A–C). The presence of CLCs was also found in the diseased ethmoid mucosa, although the positive percentage is much lower than that in NPs (data not shown), which suggesting that there is eosinophil‐dominated CRS in addition to eosinophil‐dominated NPs. We did not find any CLCs in Non‐Eos CRSwNP and control groups (Figure 1C). In addition, our data showed that CRSwNP patients with CLCs account for 84.85% (28/33) of recurrent group, whereas CRSwNP patients without CLCs account for 15.15% (5/33) of recurrent group (Table 1). As expected, patients with Eos CRSwNP account for 87.88% (29/33) of recurrent group, whereas patients with Non‐Eos CRSwNP occupy 12.12% (4/33) of recurrent group. These results suggest CLCs, similar to eosinophils, are closely associated with NPs recurrence.

**FIGURE 1:**
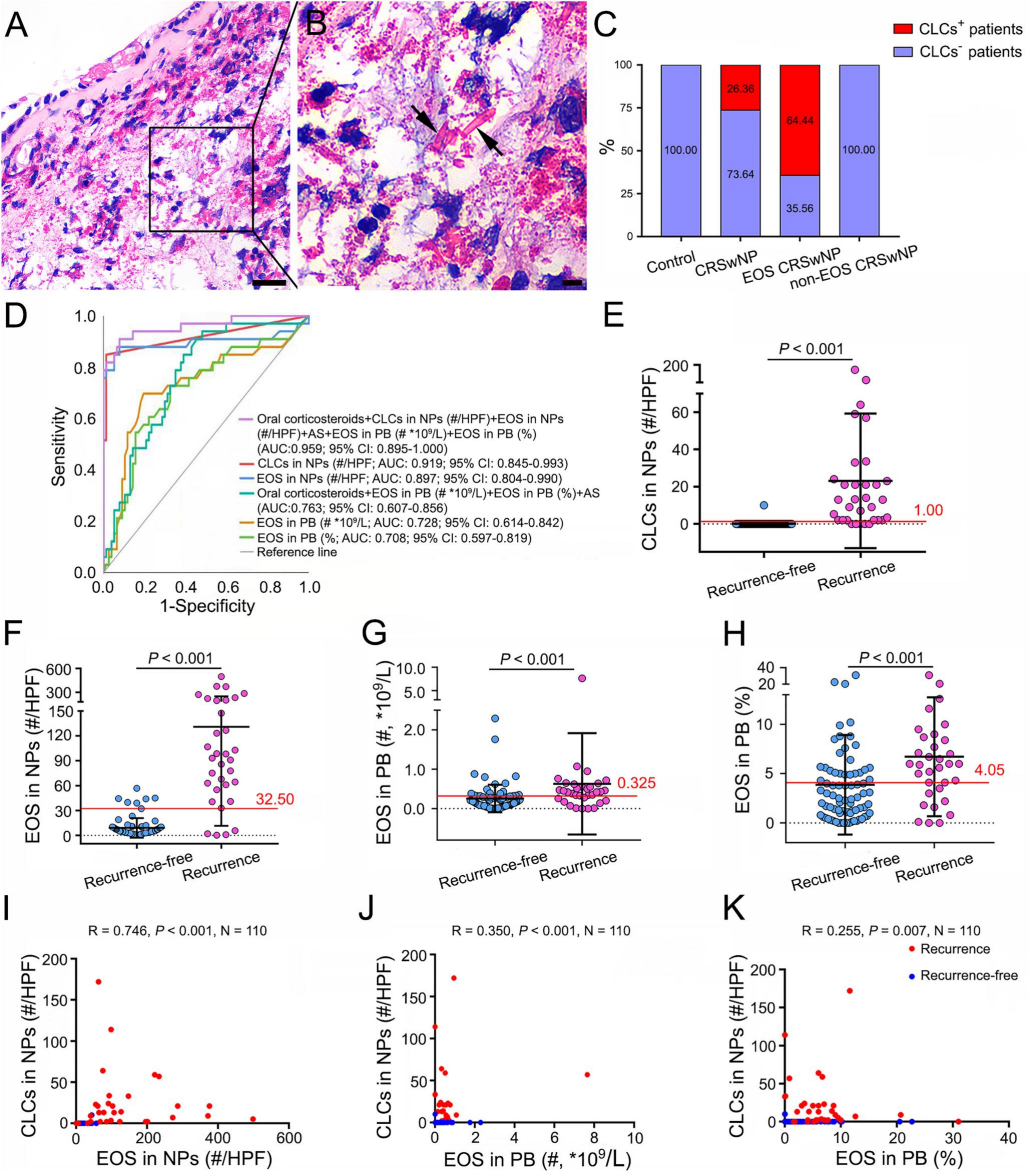
The presence of crystalline CLC structures and characteristics of predicted parameters in patients with CRSwNP. (A) Representative photomicrographs of crystalline CLC structures in patients with Eos CRSwNP detected by H&E staining (original magnification: 400×). Scale bar, 25 μm. (B) The higher magnification of the outlined area in (A). Black arrows indicate crystalline CLC structures. Scale bar, 5 μm. (C) Quantitative analysis of crystalline CLC structures in controls and CRSwNP with distinct endotypes. (D) The AUCs for the predictors of polyp recurrence. (E) The optimal cut‐off point for crystalline CLC structures in NPs to predict recurrence. (F) The optimal cut‐off point for eosinophils in NPs to predict recurrence. (G and H) The optimal cut‐off point for eosinophils count and percentage in PB to predict recurrence. (I–K) Correlation between count number of crystalline CLC structures and tissue eosinophils (I), and peripheral eosinophils (J), and percentage of peripheral eosinophils (K). AUCs, area under the curves; CLCs, Charcot‐Leyden crystals; Eos CRSwNP, eosinophilic chronic rhinosinusitis with nasal polyps; EOS, eosinophil; HPF, high power field; non‐Eos CRSwNP, non‐eosinophilic chronic rhinosinusitis with nasal polyps; NPs, nasal polyps; PB, peripheral blood.

The logistic regression analysis was employed to determine the specific factors associated with polyp recurrence. Univariable parameters were based on between‐group comparison analysis. The analysis demonstrated that tissue CLCs count (odds ratio = 2.203, 95% CI: 1.256–3.865, *p* = 0.006) was significantly associated with polyp recurrence. In addition, tissue eosinophil count (odds ratio = 1.077, 95% CI: 1.045–1.109, *p* < 0.001), male gender (odds ratio = 3.895, 95% CI: 1.330–11.403, *p* = 0.013), comorbid asthma (odds ratio = 3.974, 95% CI: 1.229–12.850, *p* = 0.021), PB eosinophil count (odds ratio = 3.804, 95% CI: 1.003–14.428, *p* = 0.049) and percentage (odds ratio = 1.096, 95% CI: 1.011–1.187, *p* = 0.025) were also identified as potential predictors for postoperative polyp recurrence. To exclude the influence of gender (male), age, comorbid allergic rhinitis, comorbid asthma, and atopic status on polyp recurrence, we performed adjusted models with multiple logistic regression, and found that CLCs are independent factors for NP recurrence (odds ratio for CLCs = 2.078, 95% CI: 1.269–3.402, *p* = 0.004).

ROC curves for the chosen parameters associated with polyp recurrence and the corresponding area under the curves (AUCs) were shown in Figure 1D. AUC values indicated that both count number of CLCs and eosinophils in NPs presented high predictive values for NP recurrence (AUC = 0.919, 95% CI = 0.845–0.993, *p* < 0.001; AUC = 0.897, 95% CI = 0.804–0.990, *p* < 0.001, respectively), whereas count number and percentage of eosinophils in PB showed relatively lower predictive values (AUC = 0.728, 95% CI = 0.614–0.842, *p* < 0.001; AUC = 0.708, 95% CI = 0.597–0.819, *p* < 0.001, respectively) compared with parameters in nasal tissues. However, there was no significant difference between the AUCs for count number of CLCs and tissue eosinophils (*Z* = 0.808; *p* = 0.419). These results demonstrated that CLCs have the similar capability as eosinophils in tissue to predict NP recurrence. Moreover, ROC curve using combination of CLCs + presence of asthma + count number and percentage of eosinophils in PB + count number of eosinophils in nasal tissue + need for oral corticosteroids (AUC = 0.959, 95% CI = 0.915–1.000, *p* < 0.001) had better sensitivity and specificity as compared to ROC curve using combination of presence of asthma + count number and percentage of eosinophils in PB + need for oral corticosteroids (AUC = 0.763, 95% CI = 0.670–0.856, *p* < 0.001). However, parameters like gender and age might not affect sensitivity and specificity of CLCs curve (data not shown).

The maximal Youden index (YI) was used to identify the optimal cut‐off point for the recurrence of NPs. The CLCs count number of 1.00 (YI = 0.835) was identified as an optimal cut‐off point for prediction of polyp recurrence with sensitivity of 84.80% and a specificity of 98.70% (Figure 1E). Similarly, the tissue eosinophil count number of 32.50 (YI = 0.801) was identified as an optimal cut‐off point for prediction of polyp recurrence with a sensitivity of 87.90% and a specificity of 92.20% (Figure 1F). Moreover, the eosinophil percentage of 4.05% (YI = 0.402) and count number of 0.325 × 10^9^/L (YI = 0.502) was also identified as the optimal cut‐off point for prediction of polyp recurrence, respectively (Figure 1G,H). The associations between CLCs and eosinophils in tissues and PB were investigated. As expected, count number of CLCs was positively correlated with eosinophils in NPs (*R* = 0.746, *p* < 0.001, Figure 1I). Moreover, CLCs count was modestly but significantly associated with the count number (*R* = 0.350, *p* < 0.001) and percentage (*R* = 0.255, *p* = 0.007) of eosinophils in PB (Figure 1J,K).

To our best knowledge, we first described the predictive value of crystalline CLC structures for NP recurrence. There are some advantages and disadvantages of measuring CLC crystal structures in nasal biopsies. First, ROC curves of CLCs and tissue eosinophils are similar, which means that in addition to eosinophils, a novel and alternative biomarker is provided for predicting nasal relapse. Second, there is a lack of unanimous histopathologic criteria and no consensus regarding a cut‐off point for eosinophilia. In addition, numbers of eosinophils are pretty extensive (even more than 500 per high power field) in nasal tissue, and counting such tons of eosinophils is time‐costing. CLCs, identified as hallmarks of hyperactivated eosinophils, could serve as the easy‐counting and objective biomarkers for predict NP recurrence. Third, since CLCs‐dissolving antibodies suppressed airway inflammation by blunting goblet‐cell metaplasia and IgE synthesis in a humanized mouse model,^9^ CLCs might have novel implications for potential therapeutic targets of CRSwNP. However, staining CLCs with H&E is a little tricky and requires an optimal pH value of eosin, otherwise small size of CLCs are inevitably missed. Frankly, we did not perform intra‐individual comparison of CLCs in NP tissue, nasal mucosa tissue and nasal secretion, so we cannot draw the conclusion which form of CLC is the best predictor for NP recurrence. It's interesting to compare the sensitivity and specificity of distinct forms of CLC with different biopsies for predicting NP relapse in the future work.

## 1 CONCLUSION

The crystalline CLC structures visualized by histomorphology in NPs are more than markers of eosinophilic inflammation, but an objective and promising predictor for NP recurrence. The crystalline CLC structures may serve as a potential target for CRSwNP treatment strategy, particularly Eos CRSwNP.

## AUTHOR CONTRIBUTIONS

All authors participated in the drafting and revision of the manuscript and interpretation of findings. Wenyi Chen performed H&E staining, data analysis and manuscript preparation. Yurong Bai did H&E staining, and figure and manuscript preparation. Weifeng Kong, Xin Luo, Yinhui Zeng, Jingyuan Chen, Xinyue Wang, Qingwu Wu participated in clinical data collection and manuscript preparation. Shuvam Chaudhuri performed data analysis and manuscript preparation. Jianning Chen contributed to histomorphological analysis and manuscript preparation. Qintai Yang and Yana Zhang designed the study, recruited the patients and prepared the manuscript.

## CONFLICTS OF INTEREST

There are no conflicts of interest to declare. All authors have approved the manuscript and agreed to submission.

## FUNDING INFORMATION

National Natural Science Foundation of China, Grant/Award Number: 82171114, U20A20399; Key‐area Research and Development Program of Guangdong Province, Grant/Award Number: 2020B0101130015; Natural Science Foundation of Guangdong Province, Grant/Award Number: 2022A1515011787; Science and Technology Program of Guangzhou, Grant/Award Number: 202201020402.
